# Anatomical Variations of the Iliohypogastric Nerve: A Systematic Review of the Literature

**DOI:** 10.7759/cureus.24910

**Published:** 2022-05-11

**Authors:** Konstantinos Manolakos, Konstantinos Zygogiannis, Chagigia Mousa, Theano Demesticha, Vasileios Protogerou, Theodore Troupis

**Affiliations:** 1 6th Orthopedic Department, KAT Hospital, Athens, GRC; 2 Department of Trauma and Orthopedics, Laiko General Hospital of Athens, Athens, GRC; 3 Department of Anatomy and Surgical Anatomy, Medical School, National and Kapodistrian University of Athens, Athens, GRC

**Keywords:** lower abdomen, ilioinguinal nerve, anatomical variations, lumbar plexus, iliohypogastric nerve

## Abstract

Several anatomical variations of the iliohypogastric nerve branches have been observed in earlier studies. Knowledge of these variations is useful for the improvement of peripheral nerve blocks and avoidance of iatrogenic nerve injuries during surgeries. The purpose of this study was to perform a systematic review of the literature about the anatomical topography and variations of the iliohypogastric nerve. An extensive search on PubMed, Scopus, and Web of Science electronic databases was conducted by the first author in November 2021, based on the Preferred Reporting Items for Systematic Reviews and Meta-Analyses (PRISMA) guidelines. Anatomical or cadaveric studies about the origin, the course, and the distribution of the iliohypogastric nerve were included in this review. Thirty cadaveric studies were included for qualitative analysis. Several anatomical variations of the iliohypogastric nerve were depicted including its general properties, its origin, its branching patterns, its course, its relation to anatomical landmarks, and its termination. Among them, the absence of the iliohypogastric nerve ranged from 0 to 34%, its origin from L1 ranged from 62.5 to 96.5%, and its isolated emergence from psoas major ranged from 47 to 94.5%. Numerous anatomical variations of the iliohypogastric nerve exist but are not commonly cited in classic anatomical textbooks. The branches of the iliohypogastric nerve may be damaged during spinal anesthesia and surgical procedures in the lower abdominal region. Therefore, a better understanding of the regional anatomy and its variations is of vital importance for the prevention of iliohypogastric nerve injuries.

## Introduction and background

The iliohypogastric nerve is the first nerve of the lumbar plexus. It derives from the anterior branch of the first lumbar nerve (L1), occasionally with the contribution from the 12th thoracic nerve (T12). In parallel to the ilioinguinal nerve, the iliohypogastric nerve emerges from the lateral border of the psoas muscle, anterior to quadratus lumborum muscle, behind the renal fossa, into the kidney fat, and behind the lower pole of the kidney. In its retroperitoneal course, the iliohypogastric nerve runs in parallel and between the subcostal nerve above and the ilioinguinal nerve below [1-2].

Approximately above the posterior third of the iliac crest, the iliohypogastric nerve penetrates the posterior fascia of the transverse abdominal muscle, courses between the transverse abdominal muscle and the internal oblique muscle, in parallel and at a distance of 1.5 cm from the iliac crest. At this point, the iliohypogastric nerve is separated into two terminal branches: an abdominal and a genital branch. Just proximal to its terminal separation, at the lateral summit of the iliac crest, the iliohypogastric nerve gives off its only collateral branch, a lateral cutaneous branch, which is distributed in the skin of the posterior gluteal region [3-4].

The abdominal (muscular) branch courses along the lateral abdominal wall between the transverse abdominis muscle and internal oblique muscle. Near the outer orifice of the inguinal canal, it pierces the internal oblique muscle and then courses between the internal oblique muscle and external oblique muscle to reach the sheath of the rectus abdominis muscle. Posterior to the rectus abdominis muscle, the abdominal branch is divided into a lateral and a medial cutaneous branch to provide sensory innervation of the suprapubic region. At its pathway, between the abdominal muscles, the abdominal branch of the iliohypogastric nerve is connected with abdominal branches from the ilioinguinal and the subcostal nerve, giving off several branches innervating the muscles of the lower part of the lateral abdominal muscles [5-6].

Along with the abdominal branch, the genital (sensory) branch of the iliohypogastric nerve runs between the transverse abdominis muscle and internal oblique muscle. At the level of the anterior superior iliac spine, it pierces the internal oblique muscle and runs in parallel and superior to the inguinal ligament. In the inguinal area, the genital ramus runs along the aponeurosis of the external oblique muscle, anterior to the internal oblique muscle. About 2-4 cm above the subcutaneous orifice of the inguinal canal, this genital branch becomes subcutaneous and gives sensory innervations to the skin of the pubic area [5-6].

Numerous anatomical variations of the origin, the course, and the distribution of iliohypogastric nerve branches have been observed in previous studies [5,7-8]. Although the general anatomy of the iliohypogastric nerve has been well documented in the literature [9], the identification of anatomical variations is of vital importance to prevent iatrogenic nerve injury during hernia surgery [10], laparoscopic surgeries [11], Pfannenstiel incisions [11], needle suspensions of the bladder [12], and gynecological procedures [13]. Knowledge of the anatomical variations of the iliohypogastric nerve is helpful in ensuring the efficacy of peripheral nerve blocks. Special attention should be paid not to include iliohypogastric nerve branches while suturing, in order to avoid postoperative neuropathies. The purpose of this study is to perform a systematic review of the literature about the anatomical topography and variations of the iliohypogastric nerve.

## Review

1. Materials and methods

An extensive search on PubMed, Scopus, and Web of Science electronic databases was conducted by the first author in November 2021, as per the Preferred Reporting Items for Systematic Reviews and Meta-Analyses (PRISMA) guidelines [14]. The keyword “iliohypogastric nerve” was used. Moreover, the reference lists of the included papers were scanned for additional studies. Anatomical or cadaveric studies about the origin, the course, and the distribution of the iliohypogastric nerve were included in this review. The exclusion criteria were as follows: (a) study protocols, case reports, systematic reviews, and meta-analyses, (b) studies in non-English language or those without available full text, and (c) non-human studies.

2. Results

**Figure 1:**
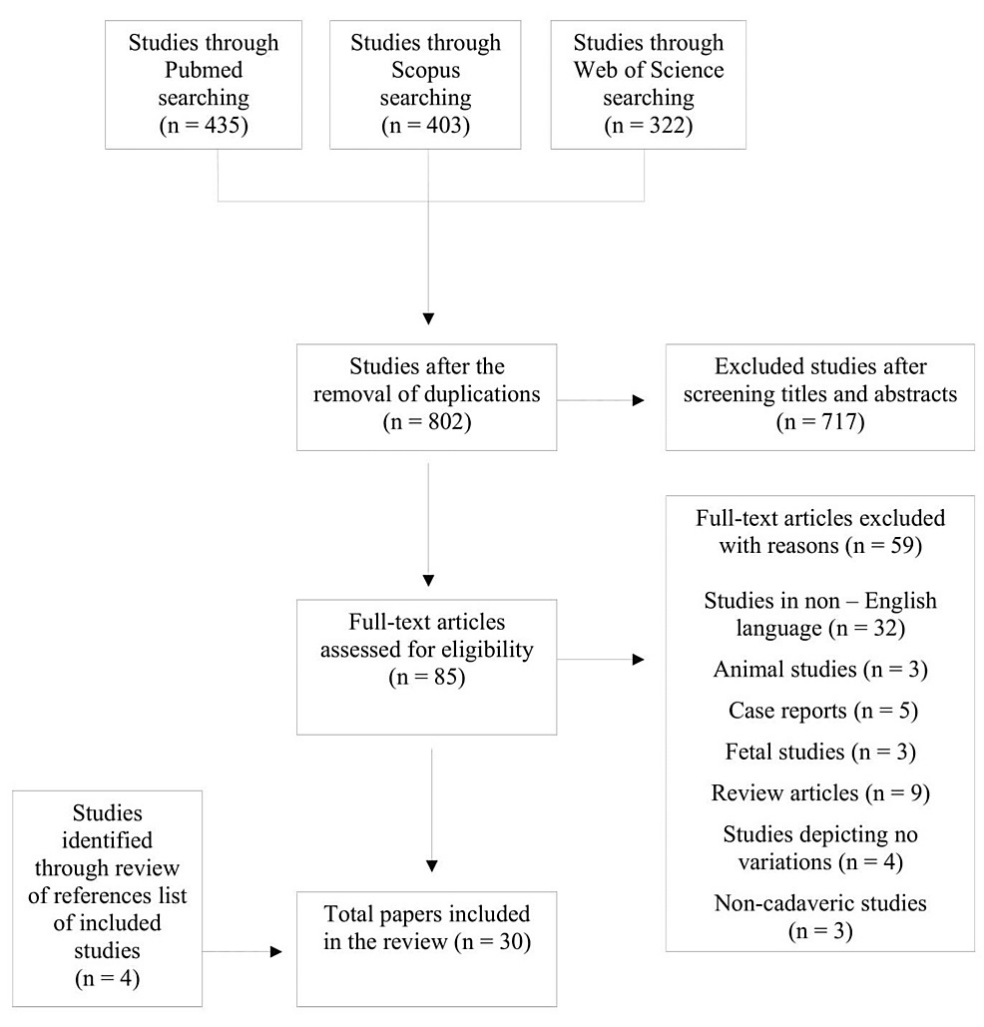
Flowchart illustrating the selection of studies

The Initial search yielded a total of 1,160 studies (Figure 1).

After the removal of duplicates, 802 studies were evaluated. After the screening of titles and abstracts, 717 were rejected, leaving 85 studies for full-text evaluation. Of them, 59 studies were excluded for the above-mentioned mentioned reasons. Moreover, four studies were added after reviewing the references list of the included studies. Finally, 30 studies were included for qualitative analysis [5,7-8,11,15-40].

2.1. General Properties

As shown in Table 1, the reported absence of the iliohypogastric nerve in cadaveric studies ranged from 0 to 34.35%.

**Table 1:** Reported frequency of absence of the iliohypogastric nerve N: number of iliohypogastric nerves studied

Study (year)	N	Rate of absence of the iliohypogastric nerve
Moreno-Egea (2021) [25]	100	1%
Paul and Shastri (2019) [39]	60	6.6%
Gogi (2019) [38]	40	0%
Anandhi et al. (2018) [36]	50	2%
Arora et al. (2016) [37]	60	13.34%
Nontasaen et al. (2016) [28]	131	34.35%
Geh et al. (2015) [20]	43	0%
Gandhi et al. (2013) [19]	60	0%
Anloague and Huijbregts (2009) [16]	38	20.6%
Wijsmuller et al. (2006) [34]	18	0%

According to Maigne et al., the cutaneous branch of the iliohypogastric nerve is absent in 10% of cases [35]. When the iliohypogastric nerve is absent, its function may be taken over by the genitofemoral nerve [39]. One study has reported the existence of a double iliohypogastric nerve with a 1.66% frequency. Two studies have reported the mean width of the iliohypogastric nerve. Klaasen et al. calculated the mean diameter of the iliohypogastric nerve to be 2 mm (range: 1-2.8 mm) [24]. Similarly, the calculated mean width by Izci et al. was 2.2 mm (range: 2.0-2.5 mm) [21].

2.2. Origin

The origin of the iliohypogastric nerve has shown great variability. It is known that the iliohypogastric nerve is derived from L1 with the occasional contribution from T12 [41]. As shown in Table 2, the iliohypogastric nerve originates from L1 at reported rates ranging from 62.5 to 96.5%, followed by T12-L1, with reported rates of 0-37.5%.

**Table 2:** Variations in the origin of the iliohypogastric nerve N: number of iliohypogastric nerves studied

Study (year)	N	T11-T12	T12	T12-L1	L1	L1-L2
Ji and Hur (2021) [23]	30	0%	28.6%	0%	71.4%	0%
Paul and Shastri (2019) [39]	60	0%	0%	6.6%	83.6%	1.6%
Gogi (2019) [38]	40	0%	0%	37.5%	62.5%	0%
Anandhi et al. (2018) [36]	50	0%	2%	10%	86%	0%
Nontasaen et al. (2016) [28]	131	0%	0%	3.5%	96.5%	0%
Arora et al. (2016) [37]	60	0%	0%	8.33%	78.3%	0%
Gandhi et al. (2013) [19]	60	0%	0%	13.4%	86.6%	0%
Klaasen et al. (2011) [24]	200	6%	7%	14%	73%	0%

In rare cases, the iliohypogastric nerve may derive from T12 and T11 roots. The mean thicknesses of T12 and L1 constituting the iliohypogastric nerve have been measured to be 2.4 mm and 1.2 mm, respectively [23].

It has been reported that the iliohypogastric nerve may communicate with accessory nerve branches to subcostal, ilioinguinal, and lateral femoral cutaneous nerves [24]. In the case of a common trunk between the iliohypogastric nerve and the subcostal nerve (20% of cases), the separation of the two nerves may occur at the anterior surface of the quadratus lumborum muscle, near the origin of the transverse abdominal muscle, or laterally between the transverse abdominal muscle and the internal oblique muscle [7].

2.3. Branching Patterns

The iliohypogastric nerve may emerge from the outer border of the psoas muscle, either united with a common trunk with the ilioinguinal nerve (Type I) or, most commonly, as a separate nerve (Type II). As shown in Table 3, the classic pattern or Type II in cadavers ranges from 47 to 94.5%.

**Table 3:** Variations of the branching pattern of iliohypogastric and ilioinguinal nerves N: number of iliohypogastric nerves studied

Study (year)	N	Type I (single trunk of the two nerves)	Type II (two separate branches)
Moreno-Egea (2021) [25]	100	22%	78%
Reinpold et al. (2015) [32]	60	18%	82%
Geh et al. (2015) [20]	43	53%	47%
Gandhi et al. (2013) [19]	60	11.7%	88.3%
Klaasen et al. (2011) [24]	200	20%	80%
Ndiaye et al. (2010) [27]	100	14%	86%
Rahn et al. (2010) [40]	36	50%	50%
Anloague and Huijbregts (2009) [16]	38	5.8%	94.2%
Peschaud et al. (2006) [30]	40	7.5%	92.5%
Tubbs et al. (2005) [33]	22	25%	75%
Mandelkow and Loeweneck (1988) [7]	42	30%	70%
Papadopoulos and Katritsis (1981) [29]	348	46.83%	53.17%

In the case of Type I branching pattern, the division of the iliohypogastric and the ilioinguinal nerve may be located behind the kidney, or between the internal oblique muscle and transverse abdominal muscle [7]. According to Geh et al., in Type 1 cases, the single trunk of the two nerves had a mean distance of 2.7 ± 1.5 cm from the psoas-diaphragm junction [20]. In the study by Klaasen et al., the mean distance of the two nerves before the union was 2.6 cm (range: 1-5.5 cm) [24]. Gandhi et al. observed that the common trunk of these nerves had a mean length of 6 cm within the belly of the psoas muscle before their separation into the iliohypogastric and ilioinguinal nerve [19].

In the case of Type II branching pattern, according to Moreno-Egea, the iliohypogastric nerve emerges from the lateral border of the psoas, at a mean distance of 2.5 ± 0.8 cm (range: 1.3-4.2 cm), from the ilioinguinal nerve [25]. In the study by Geh et al., the mean distance of the iliohypogastric nerve to the psoas-diaphragm junction was 2.5 ± 1.6 cm [20]. Measured from the midline, the iliohypogastric nerve arises from the lateral border of the psoas muscle at a mean distance of 6 cm (range: 5-8 cm) [33].

2.4. Retroperitoneal Course

The iliohypogastric nerve always lies on the anterior surface of the quadratus lumborum muscle, which is the position with the minimum anatomic variability. According to Gandhi et al., the iliohypogastric nerve was 1.5 cm apart all along its course across the quadratus lumborum muscle, on the posterior abdominal wall [19]. It enters the abdominal wall above the iliac crest and lateral from the anterior superior iliac spine [32-33].

The anatomical variations of the iliohypogastric nerve increase from central to peripheral. The iliohypogastric nerve is almost always lateral to the posterior superior iliac spine [32]. According to Reinpold et al., the iliohypogastric nerve runs in almost all cases at 0.4-4.3 cm laterally and 1.0-5.3 cm cranially in relation to the posterior superior iliac spine, with a mean distance of 8.2 ± 0.8 cm (range: 5.1-9.2 cm) cranially [32]. On the contrary, Moreno-Egea observed that the iliohypogastric nerve was always cranially and medially to the posterior superior iliac spine [25].

2.5. Relation to the Anterior Superior Iliac Spine

The iliohypogastric nerve leaves retroperitoneal space and enters the abdominal wall, by penetrating the aponeurosis of the transverse abdominal muscle following a highly variable course. Table 4 shows the reported mean distances from the penetration of transverse abdominal muscle to the anterior superior iliac spine.

**Table 4:** Mean distance from the penetration of the abdominal wall to the anterior superior iliac spine N: number of iliohypogastric nerves studied; IHN: iliohypogastric nerve; TAM: transverse abdominal muscle; ASIS: anterior superior iliac spine; SD: standard deviation

Study (year)	N	Mean distance from IHN penetration of TAM to ASIS (cm) ± SD
Klaasen et al. (2011) [24]	200	2.8 ± 1.3 (range: 1.1–5.5) medial; 1.4 ± 1.2 (range: 0.6–5.1) inferior
Whiteside et al. (2003) [11]	13	2.1 ± 1.8 (range: -1.6 to 5.0) medial; 0.9 ± 2.8 (range: -5.4 to 5.5) inferior
Reinpold et al. (2015) [32]	56	6.9 ± 3.1 (range: 2.0–12.3) dorsal

The iliohypogastric nerve is always superior to the supracristal plane, with a mean distance of 4 cm [19,33]. In the lateral inter-muscular space, the iliohypogastric nerve follows a common course with the ilioinguinal nerve over 5.6-9.0 cm (mean: 7.2 ± 3 cm). This course was always 2 cm below the anterior superior iliac spine [30]. Table 5 shows the reported mean distances from the emergence of the iliohypogastric nerve in the internal oblique muscle to the anterior superior iliac spine.

**Table 5:** Mean distance from the emergence of the internal oblique muscle to the anterior superior iliac spine N: number of iliohypogastric nerves studied; IHN: iliohypogastric nerve; IOM: internal oblique muscle; ASIS: anterior superior iliac spine; SD: standard deviation

Study (year)	N	Mean distance from IHN emergence of IOM to ASIS (cm) ± SD
Rahn et al. (2010) [40]	36	2.5 (range: 0–4.6) medial; 2.0 (range: 0–4.6) inferior
Peschaud et al. (2006) [30]	40	2.8 ± 1.2 (range: 2.5–3.2) medial
Avsar et al. (2002) [17]	24	3.95 (range: 1.5–8) right; 2.86 (range: 2.3–3.6) left
Mandelkow and Loeweneck (1988) [7]	40	2.59 ± 0.73

2.6. Relation to the Inguinal Canal

Previous studies have measured the mean distance of the iliohypogastric nerve from the middle of the inguinal ligament as 2.7 cm. In 22% of cases, this distance was less than 2 cm [29]. Wijsmuller et al. showed that in 89% of cases, the iliohypogastric nerve perforated the internal oblique muscle at a mean distance of 2.4 cm cranially to the internal ring. However, in 11% of cases, the iliohypogastric nerve pierced the internal oblique muscle approximately in the middle and cranially to the spermatic cord [34]. The study by Salama et al. reported that in 82% of cases, the iliohypogastric nerve emerged in the lateral third of the inguinal ligament, in the insertion of the internal oblique muscle [5]. Subsequently, the iliohypogastric nerve coursed approximately horizontally and ventrally to the internal oblique muscle, piercing the deep fascia of the external oblique muscle at a mean distance of 3.8 cm cranially from the external inguinal ring. In 89%, the iliohypogastric nerve pierced the deep fascia of the external oblique muscle as one single branch [34]. Sometimes, the anterior branch of the iliohypogastric nerve is replaced by the ilioinguinal nerve just before the former exits from the external inguinal ring [34].

2.7. Termination

According to Salama et al., the genital branch of the iliohypogastric nerve was absent in 12% of cases. When present, these genital branches terminate at the deep surface of the fascia of the external oblique muscle. At this point, the genital branch of the iliohypogastric nerve is divided into two branches. In 95% of cases, the superior terminal (pubic) branch exits the inguinal canal by a separate opening distinct from the superficial orifice of the canal and innervates the pubic region. On the other hand, in 5% of cases, the inferior terminal branch leaves the inguinal canal either through the superficial orifice or a separate button-like opening and gives sensory innervation to femoral and scrotal regions [5]. In 60% of cases, the distal portions of the iliohypogastric and the ilioinguinal nerve are joined, forming a single genital branch [5,15,41].

The mean distance of the termination of the iliohypogastric nerve from the midline has been measured in three studies. According to Klaasen et al., the mean distance is 4 ± 1.3 cm (range: 2.0-12.6 cm) lateral [24]. Whiteside et al. measured this distance to be 3.7 ± 2.7 cm (range: 1.0-10.6 cm) lateral. Moreover, they found that the iliohypogastric nerve terminated at a mean distance of 5.2 ± 2.6 cm (range: 2.1-10.9 cm) superior to pubic symphysis [11]. The study by Rahn et al. observed that at a point 2 cm superior to the pubic symphysis, the iliohypogastric nerve was at a mean distance of 3.8 cm (range: 1.3-5.7 cm) lateral to the midline [40]. A cadaveric study by Cardenas-Towers et al. showed that a rectus abdominis fascia graft harvested 5.4 cm superior to the pubic symphysis should minimize injury to the iliohypogastric nerve [18].

3. Discussion

The anatomical variations of the iliohypogastric nerve are extensively reported in the literature. To the best of our knowledge, this is the first systematic review that attempts to record all the anatomical variations of the iliohypogastric nerve, in relation to its properties, origin, branching patterns, course, relation to anatomical landmarks, and termination.

The knowledge of the anatomical variations of the iliohypogastric nerve is critical in lower abdomen operations, regional anesthesia, and nerve entrapment syndromes. The branches of the iliohypogastric nerve are closely related to surgical approaches in the lower abdomen inferior to the superior anterior iliac spine and may be damaged at skin incisions or trauma suturing, causing nerve entrapment [7,42-43]. Damage to the iliohypogastric nerve can result in paresis of abdominal muscles and sensory deficit over the iliac crest and above the pubic symphysis.

It has been reported that the frequency of the failure of iliohypogastric nerve blockades is 10-25% [44]. Regardless of the applied technique for regional anesthesia, the origin of fibers comprising the iliohypogastric nerve and the anatomical route are of vital importance for the proper administration of local anesthetic. Needle malposition at the administration of the blockade may result in postoperative nerve injuries [19]. Therefore, optimal knowledge of the regional anatomy and the nerve anatomical variations is essential for the prevention of iatrogenic nerve injuries.

In the present review, the reported incidence of complete absence of that iliohypogastric nerve is up to 34%. Most commonly, it derives from the L1 root with or without the contribution of T12. However, in the literature, sensory fibers composing the iliohypogastric nerve have been reported to encompass a region of the spinal cord extending from T11 to L2. The nerve may emerge from the psoas major, united with the ilioinguinal nerve, but most commonly (47-94%), it arises separately as a single nerve. The two nerves may initially arise separately and communicate at the iliac crest. In such cases, the iliohypogastric nerve is typically reported to supply the missing ilioinguinal branches. All these complicated origins, interconnections, and anastomoses of the branches of the iliohypogastric nerve may result in sensory overlap or provoke chronic spontaneous neuropathies and failures and complications regarding their blockades [24,45]. This complexity of the branching patterns may modify the clinical expression of the lesions of the iliohypogastric nerve by enlarging the neuralgic area and influencing the selectivity of the nerve blockade [24].

The retroperitoneal course of the iliohypogastric nerve also contains certain variations. Studies reporting its relation to the posterior superior iliac spine have shown contradictory results [25,32]. As variability increases from the spinal cord to the terminal distribution, the most reliable area to detect the iliohypogastric nerve is on the anterior surface of the quadratus lumborum. The nerve enters the abdominal wall medially and inferiorly to the anterior iliac spine and is always superior to the supracristal plane [19,33]. In the majority of cases, the iliohypogastric nerve perforated the internal oblique muscle cranially to the internal inguinal ring and pierced the aponeurosis of the external oblique muscle cranially to the external inguinal ring [34]. At this point, the iliohypogastric nerve may unite with the ilioinguinal nerve, producing a single genital branch [5,15,41]. Finally, the iliohypogastric nerve terminates about 4 cm lateral from the midline [11,24] and 5 cm cranially to the pubic symphysis [11,18].

All the aforementioned variations of the anatomy of the iliohypogastric nerve have an embryological basis. Transcription factors, including cell surface receptors and adhesion molecules, are responsible for the embryonic development and connection of nerve fibers, by recognizing and connecting the ingredients of the extracellular matrix during neuronal growth. Several nerve growth factors are secreted from the target tissue, maintain expression of these cell adhesion molecules, and trigger the development of the axonal growth cones. Any changes in signaling between mesenchymal neuronal cells and neuronal growth cones may result in the evolution of anatomic variations [39].

## Conclusions

The results of the present review revealed numerous anatomical variations of the iliohypogastric nerve, which are not commonly cited in classic anatomical textbooks. Paying proper attention to the potential variations in the presence, the origin, the branching patterns, the course, and the distribution of the iliohypogastric nerve may prevent iatrogenic nerve injuries. The branches of the iliohypogastric nerve may be damaged during spinal anesthesia and surgical procedures in the lower abdominal region. Therefore, a better understanding of the regional anatomy and its variations is of vital importance for the prevention of iliohypogastric nerve injuries. Further studies delineating iliohypogastric nerve topography variations may improve the success rates of nerve blockades and abdominal surgical procedures and reduce the possibility of iliohypogastric nerve entrapment syndromes.

## References

[1] Mirjalili SA (2015). Anatomy of the lumbar plexus. Nerves and Nerve Injuries.

[2] Craven J (2004). Lumbar and sacral plexuses. Anaesth Intensive Care Med.

[3] Dakwar E, Vale FL, Uribe JS (2011). Trajectory of the main sensory and motor branches of the lumbar plexus outside the psoas muscle related to the lateral retroperitoneal transpsoas approach. J Neurosurg Spine.

[4] Matejcík V (2010). Anatomical variations of lumbosacral plexus. Surg Radiol Anat.

[5] Salama J, Sarfati E, Chevrel JP (1983). The anatomical bases of nerve lesions arising during the reduction of inguinal hernia. Anat Clin.

[6] Tagliafico A, Bignotti B, Cadoni A, Perez MM, Martinoli C (2015). Anatomical study of the iliohypogastric, ilioinguinal, and genitofemoral nerves using high-resolution ultrasound. Muscle Nerve.

[7] Mandelkow H, Loeweneck H (1988). The iliohypogastric and ilioinguinal nerves. Distribution in the abdominal wall, danger areas in surgical incisions in the inguinal and pubic regions and reflected visceral pain in their dermatomes. Surg Radiol Anat.

[8] Oelrich TM, Moosman DA (1977). The aberrant course of the cutaneous component of the ilioinguinal nerve. Anat Rec.

[9] Apaydin N (2015). Variations of the lumbar and sacral plexuses and their branches. Nerves and Nerve Injuries.

[10] al-dabbagh AK (2002). Anatomical variations of the inguinal nerves and risks of injury in 110 hernia repairs. Surg Radiol Anat.

[11] Whiteside JL, Barber MD, Walters MD, Falcone T (2003). Anatomy of ilioinguinal and iliohypogastric nerves in relation to trocar placement and low transverse incisions. Am J Obstet Gynecol.

[12] Miyazaki F, Shook G (1992). Ilioinguinal nerve entrapment during needle suspension for stress incontinence. Obstet Gynecol.

[13] Geis K, Dietl J (2002). Ilioinguinal nerve entrapment after tension-free vaginal tape (TVT) procedure. Int Urogynecol J Pelvic Floor Dysfunct.

[14] Moher D, Liberati A, Tetzlaff J, Altman DG (2009). Preferred reporting items for systematic reviews and meta-analyses: the PRISMA statement. PLoS Med.

[15] Akita K, Niga S, Yamato Y, Muneta T, Sato T (1999). Anatomic basis of chronic groin pain with special reference to sports hernia. Surg Radiol Anat.

[16] Anloague PA, Huijbregts P (2009). Anatomical variations of the lumbar plexus: a descriptive anatomy study with proposed clinical implications. J Man Manip Ther.

[17] Avsar FM, Sahin M, Arikan BU, Avsar AF, Demirci S, Elhan A (2002). The possibility of nervus ilioinguinalis and nervus iliohypogastricus injury in lower abdominal incisions and effects on hernia formation. J Surg Res.

[18] Cardenas-Trowers OO, Bergden JS, Gaskins JT, Gupta AS, Francis SL, Herring NR (2020). Development of a safety zone for rectus abdominis fascia graft harvest based on dissections of the ilioinguinal and iliohypogastric nerves. Am J Obstet Gynecol.

[19] Gandhi KR, Joshi SD, Joshi SS, Siddiqui AU, Jalaj AV (2013). Lumbar plexus and its variations. J Anat Soc India.

[20] Geh N, Schultz M, Yang L, Zeller J (2015). Retroperitoneal course of iliohypogastric, ilioinguinal, and genitofemoral nerves: a study to improve identification and excision during triple neurectomy. Clin Anat.

[21] Izci Y, Gürkanlar D, Ozan H, Gönül E (2005). The morphological aspects of lumbar plexus and roots. An anatomical study. Turk Neurosurg.

[22] Jacobs CJ, Steyn WH, Boon JM (2004). Segmental nerve damage during a McBurney's incision: a cadaveric study. Surg Radiol Anat.

[23] Ji HJ, Hur MS (2021). Morphometry of spinal nerve composition and thicknesses of lumbar plexus nerves for use in clinical applications. Int J Morphol.

[24] Klaassen Z, Marshall E, Tubbs RS, Louis RG Jr, Wartmann CT, Loukas M (2011). Anatomy of the ilioinguinal and iliohypogastric nerves with observations of their spinal nerve contributions. Clin Anat.

[25] Moreno-Egea A (2021). A study to improve identification of the retroperitoneal course of iliohypogastric, ilioinguinal, femorocutaneous and genitofemoral nerves during laparoscopic triple neurectomy. Surg Endosc.

[26] Ndiaye A, Diop M, Ndoye JM (2007). Anatomical basis of neuropathies and damage to the ilioinguinal nerve during repairs of groin hernias (about 100 dissections). Surg Radiol Anat.

[27] Ndiaye A, Diop M, Ndoye JM, Ndiaye A, Mané L, Nazarian S, Dia A (2010). Emergence and distribution of the ilioinguinal nerve in the inguinal region: applications to the ilioinguinal anaesthetic block (about 100 dissections). Surg Radiol Anat.

[28] Nontasaen P, Das S, Nisung C, Sinthubua A, Mahakkanukrauh P (2016). A cadaveric study of the anatomical variations of the lumbar plexus with clinical implications. J Anat Soc India.

[29] Papadopoulos NJ, Katritsis ED (1981). Some observations on the course and relations of the iliohypogastric and ilioinguinal nerves (based on 348 specimens). Anat Anz.

[30] Peschaud F, Malafosse R, Floch-Prigent PL, Coste-See C, Nordlinger B, Delmas V (2006). Anatomical bases of prolonged ilio-inguinal-hypogastric regional anesthesia. Surg Radiol Anat.

[31] Rab M, Ebmer And J, Dellon AL (2001). Anatomic variability of the ilioinguinal and genitofemoral nerve: implications for the treatment of groin pain. Plast Reconstr Surg.

[32] Reinpold W, Schroeder AD, Schroeder M, Berger C, Rohr M, Wehrenberg U (2015). Retroperitoneal anatomy of the iliohypogastric, ilioinguinal, genitofemoral, and lateral femoral cutaneous nerve: consequences for prevention and treatment of chronic inguinodynia. Hernia.

[33] Tubbs RS, Salter EG, Wellons JC 3rd, Blount JP, Oakes WJ (2005). Anatomical landmarks for the lumbar plexus on the posterior abdominal wall. J Neurosurg Spine.

[34] Wijsmuller AR, Lange JF, Kleinrensink GJ (2007). Nerve-identifying inguinal hernia repair: a surgical anatomical study. World J Surg.

[35] Maigne JY, Maigne R, Guérin-Surville H (1986). Anatomic study of the lateral cutaneous rami of the subcostal and iliohypogastric nerves. Surg Radiol Anat.

[36] Anandhi PG, Alagavenkatesan VN, Pushpa Pushpa, Shridharan P (2013). A study to document the formation of lumbar plexus, its branching pattern, variations and its relation with psoas major muscle. Int J Contemp Med Res.

[37] Arora D, Trehan SS, Kaushal S, Chhabra U (2016). Morphology of lumbar plexus and its clinical significance. Int J Anat Res.

[38] Gogi P (2019). A study of variations in iliohypogastric and ilioinguinal nerves in human adults. Int J Anat Res.

[39] Paul L, Shastri D (2019). Anatomical variations in formation and branching pattern of the border nerves of lumbar region. Nat J Clin Anat.

[40] Rahn DD, Phelan JN, Roshanravan SM, White AB, Corton MM (2010). Anterior abdominal wall nerve and vessel anatomy: clinical implications for gynecologic surgery. Am J Obstet Gynecol.

[41] Moosman DA, Oelrich TM (1977). Prevention of accidental trauma to the iloinguinal nerve during inguinal hernoirrhaphy. Am J Surg.

[42] Amin N, Krashin D, Trescot AM (2016). Ilioinguinal and iliohypogastric nerve entrapment: abdominal. Peripheral Nerve Entrapments: Clinical Diagnosis and Management.

[43] Cardosi RJ, Cox CS, Hoffman MS (2002). Postoperative neuropathies after major pelvic surgery. Obstet Gynecol.

[44] van Schoor AN, Boon JM, Bosenberg AT, Abrahams PH, Meiring JH (2005). Anatomical considerations of the pediatric ilioinguinal/iliohypogastric nerve block. Paediatr Anaesth.

[45] Weltz CR, Klein SM, Arbo JE, Greengrass RA (2003). Paravertebral block anesthesia for inguinal hernia repair. World J Surg.

